# Hypometabolism, Alzheimer’s Disease, and Possible Therapeutic Targets: An Overview

**DOI:** 10.3390/cells12162019

**Published:** 2023-08-08

**Authors:** Snehal Raut, Aditya Bhalerao, Michael Powers, Minelly Gonzalez, Salvatore Mancuso, Luca Cucullo

**Affiliations:** 1 Department of Foundational Medical Studies, Oakland University William Beaumont School of Medicine, Rochester, MI 48309, USA; sraut@oakland.edu (S.R.); abhalerao@oakland.edu (A.B.); mkgonzalez@oakland.edu (M.G.); mancuso@oakland.edu (S.M.); 2 Department of Biological and Biomedical Sciences, Oakland University, Rochester, MI 48309, USA; michaelpowers@oakland.edu

**Keywords:** glucose transporter, blood–brain barrier, Alzheimer’s disease, glucose metabolism, oxidative stress, glucose uptake

## Abstract

The brain is a highly dynamic organ that requires a constant energy source to function normally. This energy is mostly supplied by glucose, a simple sugar that serves as the brain’s principal fuel source. Glucose transport across the blood–brain barrier (BBB) is primarily controlled via sodium-independent facilitated glucose transport, such as by glucose transporter 1 (GLUT1) and 3 (GLUT3). However, other glucose transporters, including GLUT4 and the sodium-dependent transporters SGLT1 and SGLT6, have been reported in vitro and in vivo. When the BBB endothelial layer is crossed, neurons and astrocytes can absorb the glucose using their GLUT1 and GLUT3 transporters. Glucose then enters the glycolytic pathway and is metabolized into adenosine triphosphate (ATP), which supplies the energy to support cellular functions. The transport and metabolism of glucose in the brain are impacted by several medical conditions, which can cause neurological and neuropsychiatric symptoms. Alzheimer’s disease (AD), Parkinson’s disease (PD), epilepsy, traumatic brain injury (TBI), schizophrenia, etc., are a few of the most prevalent disorders, characterized by a decline in brain metabolism or hypometabolism early in the course of the disease. Indeed, AD is considered a metabolic disorder related to decreased brain glucose metabolism, involving brain insulin resistance and age-dependent mitochondrial dysfunction. Although the conventional view is that reduced cerebral metabolism is an effect of neuronal loss and consequent brain atrophy, a growing body of evidence points to the opposite, where hypometabolism is prodromal or at least precedes the onset of brain atrophy and the manifestation of clinical symptoms. The underlying processes responsible for these glucose transport and metabolic abnormalities are complicated and remain poorly understood. This review article provides a comprehensive overview of the current understanding of hypometabolism in AD and potential therapeutic targets.

## 1. Brain Energetics

The brain requires a constant supply of energy to carry out essential activities such as neurotransmission, protein synthesis, and maintenance of membrane potentials [1,2]. Most of the energy consumed by the brain is derived from glucose metabolism, which is tightly regulated to ensure adequate energy delivery to the brain [3]. Glucose is transported into the brain across the BBB and taken up by neurons and astrocytes. It is converted into ATP through biochemical reactions involving glycolysis, the tricarboxylic acid cycle (TCA), and oxidative phosphorylation [4], which is used by the brain to support a variety of cellular processes. The brain produces 95% or more of its ATP through glucose metabolism. The neurovascular unit, which includes the brain microvascular endothelial cells, pericytes [5], astrocytes, oligodendrocytes [6], microglia [7], and neurons [8] as the final recipient of glucose absorption, coordinates glucose uptake among a variety of cell types inside the brain [9]. The brain can utilize other energy sources, such as ketones, and lactate, in the absence of glucose [10,11,12].

The energy requirements of active neurons, not the blood glucose level, determine how much glucose is taken up by the brain from the bloodstream. Due to the increased energy requirements for neuronal signaling, neurotransmitter release, ionic balance, and synaptic plasticity [13,14,15,16], glucose is actively “drawn” into the brain [17,18]. Glucose transport is accomplished by the coordinated activation of glucose transporters on the capillary endothelium (GLUT1) [19,20,21,22,23,24,25], astrocytes (GLUT1 [26], GLUT2 [27], and GLUT7 [28]), oligodendrocytes (GLUT1) [29], and neurons (GLUT3 [28,30] and GLUT4) [26,31,32,33,34]. Blood glucose diffuses either directly from capillaries to neurons via the extracellular space, or is mediated by the astrocyte end-feet surrounding the capillary walls [25,35,36,37]. The distribution of energy and metabolic support inside the brain is greatly aided by astrocytes. They have extensive processes that wrap around blood vessels and synapses, allowing them to take up nutrients such as glucose and lactate from the bloodstream and deliver them to neurons.

The astrocyte–neuron lactate shuttle is a metabolic link between astrocytes and neurons. Astrocytes absorb glucose and use it to fuel glycolysis, which produces lactate as a byproduct. Subsequently, monocarboxylate transporter 1 (MCT1) transports lactate to the extracellular environment, where it is taken up by MCT2 and delivered to neurons [38]. Neurons can use lactate as a source of energy once astrocytes have released it and taken it up. This metabolic coupling supports neuronal function and plasticity by providing neurons with an extra energy source.

Unlike astrocytes and oligodendrocytes, microglia do not directly fuel neurons, although the large quantities of lactate generated by active microglia are very likely to be absorbed by nearby neurons [39]. The high energy requirements of activated microglia significantly restrict the energy available to neurons when the brain glucose supply remains limited for an extended time [40,41]. One example is during episodes of hypoglycemia [42], which can occur due to various reasons, including diabetes [43], starvation/fasting [44], critical illness [45], alcohol consumption [46], etc. When blood glucose levels drop significantly the brain’s glucose supply becomes limited, potentially leading to confusion, dizziness, seizures, loss of consciousness, and, in severe cases, brain damage. Therefore, the opportunistic use of alternate fuels derived from sources outside the brain plays a significant role in the brain’s resilience in the face of energetic strain. The primary substitute fuels for glucose are ketones and lactate, which are transported to the brain via monocarboxylate transporters on astrocytes and the capillary endothelium [47,48,49,50,51].

The brain can use ketone bodies for energy when blood levels of ketones rise sufficiently, typically after several days of fasting or after several weeks of adherence to a low-carbohydrate and high-fat (ketogenic) diet [52,53]. Fatty acid oxidation (FAO) can provide an alternative fuel source in the brain when glucose metabolism is impaired or in hypometabolic conditions [54,55]. During FAO, long-chain fatty acids are transported into the mitochondria, undergoing a series of enzymatic reactions. These reactions involve sequential cleavage of the fatty acid chain, producing acetyl-CoA units. Acetyl-CoA is then further metabolized through the TCA cycle [56,57].

**Figure 1:**
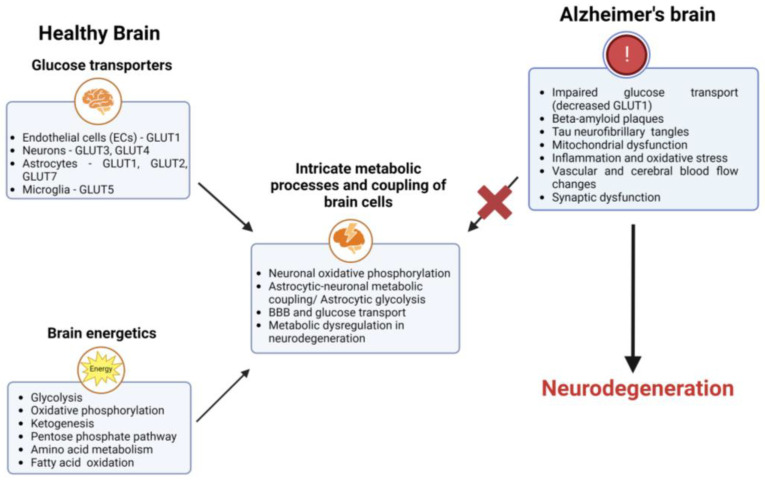
Healthy brain and brain with AD. The neurovascular unit comprises the brain’s microvascular endothelial cells, pericytes, astrocytes, oligodendrocytes, and neurons. Glucose transport is accomplished by the coordinated activation of glucose transporters on the capillary endothelium, astrocytes, oligodendrocytes, and neurons. After being transported, it is converted into ATP through biochemical reactions involving glycolysis, the TCA, and oxidative phosphorylation. It is utilized in many other energy pathways to support a variety of intricate cellular processes. Brain energetics has been implicated in various neurological disorders, including AD, as it leads to neurodegeneration and neuronal death.

Dysregulation of brain energetics has been implicated in various neurological disorders, including Alzheimer’s disease, highlighting the importance of understanding the mechanisms involved in brain energy metabolism [58,59,60,61] (Figure 1).

## 2. Hypometabolism in AD

Amyloid and tau are well-established AD risk factors that affect millions of people globally [62,63]. One of the main findings in the brains of AD patients is hypometabolism, a reduction in the brain’s metabolic rate [64,65]. This hypometabolism is seen in specific brain regions, including the temporal and parietal lobes, which are involved in memory and cognition [66,67,68,69,70]. The data from animal models and AD patients are consistent with the findings that brain hypometabolism is a major feature of AD [64,71,72]. In animal models of AD, there is a widespread decrease in the cerebral metabolic rate of glucose (CMRglu) in the hippocampus, cortex, and other brain regions [73,74,75]. This decline in glucose metabolism occurs progressively, worsening over time. Decrease in CMRglu can lead to neuronal death and cognitive decline [76,77]. While the exact reason for this hypometabolism is not well understood, it may be related to the accumulation of amyloid plaques and neurofibrillary tangles [78]. Clinical fluorodeoxyglucose (FDG) positron emission tomography (PET) studies have repeatedly demonstrated impaired glucose uptake in individuals with mild cognitive impairment (MCI), early AD, and genetic risk/family history of AD [79,80,81,82]. In AD, studies have shown that reductions in brain glucose metabolism occur early in the disease process before significant memory impairment is observed [78,83,84,85]. Additionally, neurodegeneration and loss of neurons may contribute to the decline in glucose uptake and utilization found in AD [86,87].

The exact relationship between glucose metabolism and AD is not yet fully understood. One theory is that the reduction in glucose metabolism may lead to decreased brain energy production, affecting nerve cells’ normal functioning and contributing to brain damage [88]. There is evidence that changes in insulin signaling may play a role in reducing glucose metabolism in AD [89,90]. However, research shows that glucose uptake in the brain is largely independent of insulin and that only a small percentage of glucose uptake in the brain is insulin-dependent [91,92,93], as glucose transport into the brain is primarily mediated by glucose transporters (GLUTs), which are insulin-independent [94]. While insulin can regulate glucose metabolism and uptake in other tissues in the body, it appears to have a limited role in the brain [95,96]. Research suggests that insulin may inhibit glucose uptake in the brain under certain conditions. For example, in individuals with type 2 diabetes, where insulin resistance is present, glucose uptake in the brain is not significantly impaired [97,98]. However, the reduction in glucose metabolism in certain brain regions in AD suggests that it may play a role in the disease’s development; thus, targeting glucose metabolism may provide therapeutic benefits in AD [99].

## 3. Glucose Transporters in AD

Glucose transporters play a critical role in regulating glucose uptake into the brain, and their expression levels have been investigated in AD. Here are several key findings related to glucose transporters expression in AD:

1. GLUT1 is the primary glucose transporter in the brain, and its expression is reduced in the hippocampus and frontal cortex of AD patients compared to healthy controls [100,101,102]. This reduction in GLUT1 expression may contribute to the impaired glucose metabolism observed in AD [103,104].
2. GLUT3 is another glucose transporter highly expressed in the brain, particularly in neurons [105]; studies have shown that GLUT3 expression is decreased in AD patients’ hippocampus and parietal cortex, which may contribute to the impaired glucose metabolism seen in AD [106,107].
3. GLUT4 is typically found in muscle and adipose tissue; however, recent studies have suggested that it may be expressed in the brain as well [108,109,110]. One study found that in AD patients GLUT4 expression was increased in the hippocampus, suggesting that it may be upregulated in response to the impaired glucose metabolism in this region [111,112,113].

These studies suggest that alterations in these glucose transporters’ expression may impair glucose metabolism in AD. However, this reduced expression/activity of glucose transporters in AD is likely multifactorial, resulting from a complex interplay of beta-amyloid toxicity, inflammation, and oxidative stress in the brain. Indeed, one possible explanation is that beta-amyloid plaques can directly impair the function of glucose transporters. Studies have shown that beta-amyloid can bind to these transporters, promoting their internalization and reducing glucose transport activity [101,114,115,116], thereby leading to energy deficits that contribute to the cognitive decline in AD. Another possible explanation is that inflammation, a common AD feature, can reduce expression of glucose transporters.

**Figure 2:**
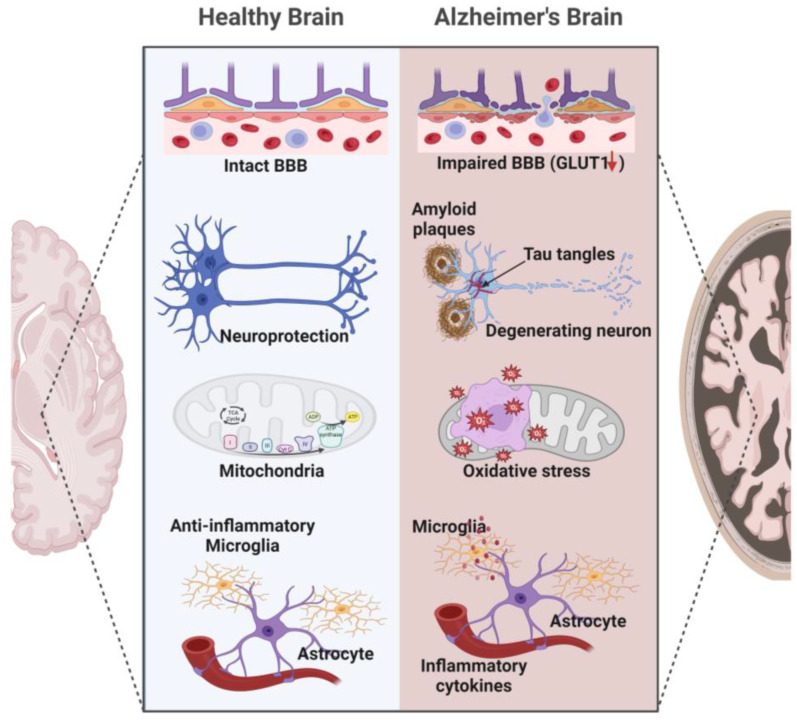
Neuropathological hallmarks of AD. The neuropathological hallmarks of AD include lesions such as amyloid plaques and neurofibrillary tangles, neuronal and synaptic loss, oxidative stress, hyperactivated glial responses leading to neuroinflammation, and BBB leakage.

Inflammatory cytokines such as interleukin-1 beta (IL-1β) and tumor necrosis factor-alpha (TNF-α) have been shown to decrease glucose transporter expression in brain cells [117,118]. Finally, oxidative stress, which is increased in AD, can severely impact the membranes of brain cells, and promote irreversible covalent modifications that destabilize and inactivate the embedded proteins, including glucose transporters [119,120,121] (Figure 2).

## 4. The Potential Therapeutic Targets for Hypometabolism in AD

Studies on AD have revealed that changes in brain glucose metabolism early in the disease process lead to the loss of synapses and neuronal death. Insufficient neuronal glucose and mitochondrial energy production compromise the clearance of amyloid and tau proteins from the brain [122]. On the other hand, the buildup of amyloid plaques and tau tangles exacerbates brain glucose hypometabolism and mitochondrial impairment, reduces energy output, and elevates oxidative stress [123,124]. Hypometabolism then causes cellular injury and neuroinflammation. This destructive cycle caused by energy failure in AD is comparable to the brain circuit disruption found in other neurodegenerative illnesses; it worsens memory and cognition and leads to unusual behavior in those affected.

While beta-amyloid is undoubtedly a major factor in the progression of AD, it is becoming increasingly obvious that a variety of other factors, such as inflammation, oxidative stress, dysfunction of the innate immune system in the brain, hereditary factors, and lifestyle choices all play a role [122,123,124]. Studies have shown that AD is associated with decreased glucose uptake and utilization in the brain, which is thought to contribute to the decline in brain function observed in this condition [125,126,127,128].

As mentioned, the conventional thinking that reduced brain glucose metabolism in AD is just a byproduct of neuronal dysfunction is now being challenged; particularly in AD, a continuous energy gap in the brain results from the gradual loss of brain glucose uptake and metabolism, resulting in brain cell malfunction and the buildup of neurotoxic proteins long before clinical symptoms become apparent. The brain’s energy demands cannot be resolved only by raising blood glucose concentration if glycolysis is compromised and neuronal function deteriorates. Furthermore, neuronal activities, not blood glucose levels, determine brain glucose absorption. Conversely, the availability of ketones and lactate in the bloodstream influences their absorption by the brain, making them an alternate source of brain energy. Brain energy rescue strategies may need to target several metabolic pathways and activities depending on the condition, because AD does not have a single common mechanism that results in hypometabolism.

Rescue strategies could focus on a single enzyme, transporter, or metabolite. One approach involves targeting the mitochondrial dysfunction associated with hypometabolism in AD. Several potential drugs that target mitochondrial function, such as coenzyme Q10, have been investigated as potential treatments for AD [129,130]. Mixed results have been found in research examining the possible therapeutic benefits of coenzyme Q10 in AD. Studies have found that CoQ10 supplementation can improve cognitive function, slow down the progression of AD, and reduce oxidative stress in individuals with the disease. Additionally, research has shown that CoQ10 can enhance mitochondrial function [130], which plays a crucial role in energy production and neuronal health. While several studies have reported positive results, others have not seen significant changes [131,132]. These studies have failed to demonstrate improvements in cognitive function or disease progression compared to a placebo or standard treatment. For instance, a large randomized controlled trial called the IDEAL Study did not find any significant differences in cognitive decline between the CoQ10-treated group and the placebo group over a two-year period [130,133]. Another approach involves targeting oxidative stress, which is believed to contribute to hypometabolism in AD [134]. Antioxidant therapies such as vitamin E and curcumin have been investigated in clinical trials as a potential treatment to reduce oxidative stress and improve cognitive function in patients with AD [135,136,137]. Vitamin E is a fat-soluble antioxidant shown to have neuroprotective effects in animal models of AD. Clinical trials have suggested that vitamin E may be beneficial in slowing cognitive decline in patients with mild to moderate AD [138,139,140]. In addition, vitamin E is a widely available and inexpensive supplement that has relatively few side effects. However, high doses of vitamin E have been associated with an increased risk of mortality and bleeding, and may have negative interactions with other medications [139]. In addition, clinical trials of vitamin E have failed to show significant cognitive benefits in patients with AD [139]. Curcumin is a naturally occurring turmeric compound with antioxidant, anti-inflammatory, and neuroprotective properties. Studies have suggested that curcumin may improve cognitive function and reduce amyloid beta and tau pathology in animal models of AD [141,142,143,144]. In addition, curcumin is a relatively safe supplement that has few side effects and low bioavailability, meaning that it is poorly absorbed by the body and quickly metabolized [145,146]. This has limited its effectiveness in clinical trials, as it is difficult to achieve therapeutic levels of curcumin in the brain. Curcumin may interact with certain medications and may negatively affect the liver in high doses [147,148,149].

In addition, growing evidence suggests that targeting the BBB can improve glucose transport and metabolism in the brain [150,151]. Disruption of the BBB is a common feature of AD, and targeting the BBB to improve glucose delivery to the brain could have therapeutic implications for AD [20,152,153]. Studies have shown a reduction of glucose transporter expression at the BBB endothelium [102,154,155]. In AD, the reduction in glucose transporter expression and distribution primarily affects the GLUT1 and GLUT3 transporters. These transporters facilitate the movement of glucose from the bloodstream into brain cells. They are integral membrane proteins that undergo conformational changes to transport glucose in a concentration-dependent manner. The expression and activity of these transporters are tightly regulated to maintain glucose homeostasis in the brain. Disruptions in the expression or function of GLUT1 and GLUT3 can lead to impaired glucose transport across the BBB, resulting in reduced glucose availability and compromised energy metabolism in the brain.

As discussed previously, a reduction in expression may result from factors such as beta-amyloid toxicity, inflammation, and oxidative stress. Targeting the disrupted BBB can help to normalize the expression and function of glucose transporters, improving the transport of glucose across the barrier and into the brain, thereby clearing amyloid-beta, modulating inflammatory responses, and preserving the integrity of the neurovascular unit [20,154,156]. Several of the cited studies discuss strategies to restore BBB integrity, such as by modulating neuroinflammation, enhancing pericyte function, and promoting tight junction integrity. Additionally, altered distribution or localization of glucose transporters within the cell membrane may contribute to impaired glucose uptake in AD. In AD, GLUT1 expression is reduced in regions such as the hippocampus and frontal cortex [28,100,157,158]. This reduction in expression may impair glucose uptake from the bloodstream into the brain. In addition, studies have reported reduced expression of GLUT3 in the hippocampus and parietal cortex. Reduced expression of GLUT3 can lead to decreased glucose uptake in neurons and contribute to energy deficits in the affected brain regions. However, GLUT1 has remained largely unexplored as a possible therapeutic target to alleviate cerebrovascular dysfunction and subsequent AD-associated neurodegeneration. Upregulating the expression of GLUTs at the BBB level (maintaining the apical versus basolateral distribution ratio) or brain cells can increase glucose flux into the brain [159]. This can be achieved through drugs or gene therapy [160].

In terms of drug-based approaches, several drugs have been investigated for their potential to upregulate GLUT expression and enhance glucose uptake in the brain. These drugs typically act through various mechanisms to increase the expression, trafficking, or activity of glucose transporters. Examples include:

1. Insulin sensitizer drugs: insulin resistance has been associated with reduced GLUT expression and impaired glucose uptake in the brain. Insulin sensitizer drugs such as thiazolidinediones (TZDs) or peroxisome proliferator-activated receptor gamma (PPARγ) agonists have been shown to enhance GLUT expression and glucose transport in the brain [161].
2. AMP-activated protein kinase (AMPK) activators: AMPK is an enzyme in cellular energy regulation. Activating AMPK can increase GLUT expression and glucose uptake. Compounds that activate AMPK, such as metformin, have been investigated for their potential to enhance brain glucose metabolism and improve cognitive function [134].
3. Other potential drugs: other drugs, including histone deacetylase inhibitors (HDAC inhibitors) [162,163], brain-derived neurotrophic factor (BDNF) [164,165], and certain antioxidants, have been studied for their ability to modulate GLUT expression and improve glucose metabolism in the brain [166].

Gene therapy approaches are another option; these involve introducing specific genes into brain cells to enhance GLUT expression [167,168]. This can be achieved through viral vectors or other delivery systems. The introduced genes can encode specific GLUT isoforms or other factors that regulate GLUT expression. Gene therapy strategies can be targeted to brain endothelial cells at the BBB or directly to brain cells [169]. However, gene therapy for brain disorders, including AD, currently remains experimental and requires further research and development [170].

## 5. The Current Stage of AD Treatments: Targeting the Usual Culprits

The neuropathological characteristics of AD include widespread deposition of Aβ plaques in the neocortex and a hierarchically ordered network of neurofibrillary tangles primarily made up of tau aggregates in the limbic and cortical association regions. According to the amyloid hypothesis, the buildup of beta-amyloid protein in the brain is a major contributor to the onset of AD [62,171]. Amyloid-beta precursor protein (APP) is a protein normally found in the membranes of neurons. Nonamyloidogenic processing of APP involves α-secretase followed by γ-secretase. In AD, APP is cleaved by two enzymes, beta-secretase (BACE1) and gamma-secretase, to form amyloid beta peptides. These peptides have the potential to accumulate into plaques in the spaces between neurons, which are observed in the brains of AD patients [172,173]. These plaques are thought to disrupt communication between neurons and contribute to the death of brain cells [174,175]. This process is thought to involve the activation of the immune system along with inflammation and oxidative stress [122,176,177].

On the other hand, the tau hypothesis focuses on the function of the tau protein in AD. Under normal conditions, this protein aids in preserving the organization of the brain’s nerve cells. Tau helps to stabilize the structure of neurons by supporting the microtubules that form the neuron’s internal transport system. Neurofibrillary tangles are aberrant tau protein clusters present in AD [123]. These tangles and amyloid plaques prevent nerve cells from operating normally, which causes brain damage and cognitive deterioration. This is fueling a growing interest in developing targeted therapies [124,125,128]. Although tangles are more closely related to neuronal loss and clinical symptoms, genetic studies implicate Aβ as a critical disease initiator for autosomal-dominantly inherited AD. These studies show that mutations in APP or enzymes that produce Aβ cause AD [63,126,127,178].

The amyloid beta peptides can bind to various receptors in the brain, including N-methyl-D-aspartate (NMDA), which plays a key role in learning and memory, Alpha7 nicotinic acetylcholine receptor (α7nAChR), which is involved in the regulation of neurotransmitter release, and receptor for advanced glycation end products (RAGE), which is involved in the immune response [179,180,181]. Tau proteins have been shown to bind to several receptors, including the low-density lipoprotein receptor-related protein 1 (LRP1) [182,183] and the sortilin-related receptor (SORL1) [184,185,186]. These receptors are involved in the transport of proteins within cells, and are thought to play a role in the clearance of tau from the brain [187]. The enzymes and genes involved in the production and clearance of amyloid beta and tau and their respective receptors have been extensively investigated as potential therapeutic targets for AD [188]. There have been several medications developed for Alzheimer’s disease that have passed through clinical trials; however, none have demonstrated significant clinical benefits. Bapineuzumab is a monoclonal antibody that targets amyloid beta plaques [189,190]. Developed by Pfizer and Johnson & Johnson, it went through several clinical trials, ultimately failing to show significant cognitive benefits in patients with Alzheimer’s disease. Solanezumab is another monoclonal antibody that targets amyloid beta plaques. Developed by Eli Lilly, it underwent several clinical trials without demonstrating significant cognitive benefits in patients with mild to moderate Alzheimer’s disease [191,192]. Crenezumab is a monoclonal antibody that targets amyloid beta plaques. It was developed by Genentech and went through several clinical trials, demonstrating no significant cognitive benefits in patients with mild to moderate Alzheimer’s disease [193,194,195]. Semagacestat is a gamma-secretase inhibitor developed by Eli Lilly. Designed to block the production of amyloid beta peptides, it ultimately failed in clinical trials due to side effects and lack of clinical benefit [196,197,198].

A few monoclonal antibodies targeting beta-amyloid or drugs aimed at blocking gamma-secretase (involved in beta-amyloid synthesis) have managed to advance through phase I and II clinical trials; however, none of these have been successful in phase III [199,200]. Furthermore, several tau protein-targeting medications, despite being expected to enhance cognitive or functional outcomes, have been unsuccessful:

1. LMTX (LMTM) is a small molecule that targets tau protein aggregation. It was evaluated in clinical trials for both AD and frontotemporal dementia (FTD). However, the Phase III trials of LMTX in AD did not significantly improve cognitive or functional outcomes compared to the placebo [201]. The reasons for this lack of efficacy are currently being investigated.
2. Tideglusib is a glycogen synthase kinase-3 beta (GSK-3β) inhibitor that can indirectly affect tau phosphorylation. GSK-3β is an enzyme involved in the abnormal phosphorylation of tau protein. Clinical trials of tideglusib in AD showed mixed results. In a Phase II trial it did not significantly affect cognitive decline [202], and a Phase III trial was discontinued due to lack of efficacy (NCT02579252).

Carriers of Aβ-enhancing genetic forms of AD (i.e., mutant APP and PS1/2, E4 variant of apolipoprotein E (APOE4) and Down’s syndrome), in which Aβ accumulates earlier in the disease, are associated with a dramatic hastening in the age of onset, with a comparable rate of progression of clinical symptoms relative to sporadic illness. This indicates that there are “Aβ-dependent” and “Aβ-independent” illness stages that respectively affect the age of initiation and the rate of progression. If this is the case, anti-Aβ treatments may not impact the outcome measure used in current AD clinical studies, which is the rate of progression after symptoms appear. The difficulties in creating effective AD treatments outline the complexity and heterogeneity of AD and the need for more focused research in order to better comprehend the disease’s pathophysiology [203,204,205].

In the past few years the FDA has approved several drugs and treatments for Alzheimer’s disease. A number of the most recent are listed below.
**Drug/Treatment****MOA****Status****Conditions**Aduhelm (aducanumab)A monoclonal antibody that targets beta-amyloid plaques in the brainApproved in June 2021Mild cognitive impairment due to AD or mild ADGantenerumabA monoclonal antibody that targets beta-amyloid plaques in the brain.Phase III clinical trialsMild ADDonanemabA monoclonal antibody that targets a modified form of beta-amyloid called N3pGPhase III clinical trialsEarly ADTauvid Imaging (flortaucipir F18)A diagnostic imaging agent that targets tau proteinApproved on May 2020Detection of tau pathology in the brainLeqembi (Lecanemab)Remove sticky clumps of the toxic protein amyloid-β from the brain.Approved on 6 January 2023Alzheimer’s disease

**Figure 3:**
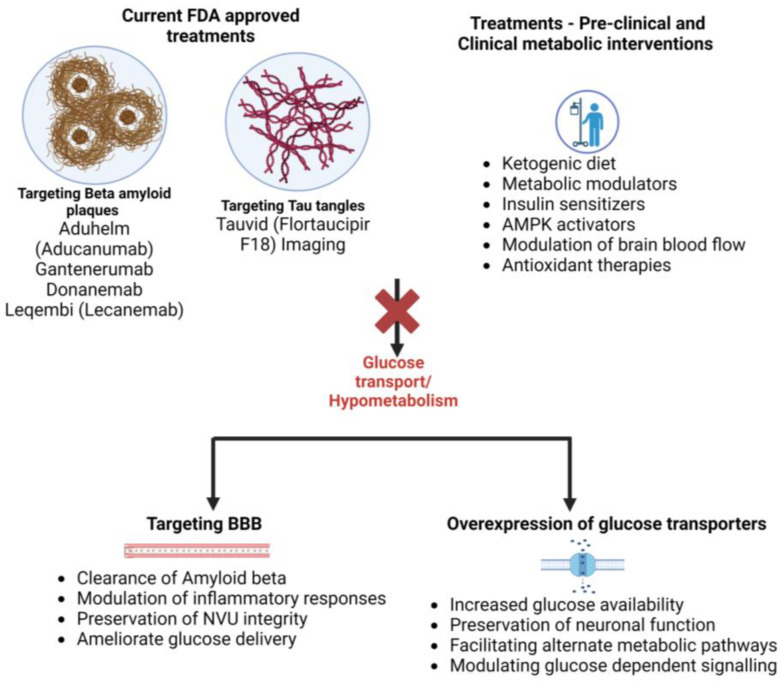
Current FDA-approved treatments and proposed targets for AD. In the past few years, the FDA has approved several drugs and treatments for AD, targeting the usual culprits, namely, beta-amyloid plaques and tau tangles. The potential therapeutic targets for hypometabolism in AD include targeting the BBB and glucose transporter overexpression.

It is important to note that while these drugs provide encouraging prospects for treating AD, much research must be undertaken in order to fully understand their effectiveness and potential risks. Additionally, many experts agree that a multifaceted approach that includes lifestyle modifications, social engagement, and cognitive stimulation is the most effective way to manage the symptoms of AD (Figure 3).

## 6. Discussion and Conclusions

While the exact cause of AD is unknown, researchers are actively exploring various avenues for treatment and prevention. These include developing new drugs to target specific pathways in the brain, using early diagnosis to start treatment earlier, and lifestyle changes such as regular exercise, a healthy diet, and mental stimulation to delay the onset of AD or slow its progression [206,207,208,209]. The amyloid and tau hypotheses provide a foundation for our understanding of the underlying causes of AD. It is a complex disorder that results from a combination of genetic, lifestyle, and medical factors. Further research is needed to develop and optimize strategies targeting glucose transport and uptake through the BBB. Nonetheless, the current evidence suggests that improving glucose delivery to the brain could provide a useful therapeutic approach to reducing the burden of AD and other neurological disorders [210]. Potential therapy approaches might try to restore the BBB’s functioning and integrity by focusing on BBB disturbance, which may indirectly affect AD glucose transport. It might be possible to improve energy metabolism and maintain neuronal function by restoring the selective permeability of the BBB, which would assist in controlling the passage of glucose and other nutrients into the brain.

Additionally, reducing the accumulation of toxic molecules through BBB repair could alleviate their negative effects on glucose transporters. It is important to note that while drug-based approaches or gene therapy strategies hold promise, several challenges are associated with upregulating GLUT expression for therapeutic purposes. The key considerations for developing such approaches include ensuring specific and targeted delivery to the brain, maintaining balanced glucose homeostasis, and avoiding potential side or off-target effects.

Hypometabolism is a decrease in the metabolic activity of brain cells and has been found to occur earlier in AD than other clinical symptoms, such as memory loss. However, hypometabolism is typically detected using advanced imaging techniques such as positron emission tomography (PET) scans, which are not routinely used in clinical practice for AD diagnosis [211,212]. Furthermore, hypometabolism alone is not specific to Alzheimer’s, and can be seen in other neurological conditions [213]. Therefore, a diagnosis of AD is typically based on a combination of clinical symptoms, imaging studies, and other tests such as neuropsychological evaluations [214].

In summary, although hypometabolism may be seen earlier in AD than other clinical symptoms, it is not considered a primary diagnostic criterion because it is not specific to this condition and requires advanced imaging techniques that are not routinely used in clinical practice. Overall, this review article highlights the growing interest in hypometabolism as a potential therapeutic target for AD and provides a comprehensive overview of the current knowledge regarding the underlying mechanisms and potential treatments. While much work remains to be done, the findings discussed in this review suggest that there are possibilities for developing new treatments for AD that target brain metabolism and related pathways.

## Data Availability

Data Availability Statements are available in the manuscript.
